# The hazards and risks of inhaled poorly soluble particles – where do we stand after 30 years of research?

**DOI:** 10.1186/s12989-019-0294-4

**Published:** 2019-02-21

**Authors:** Paul J. A. Borm, Kevin E. Driscoll

**Affiliations:** 1 Nanoconsult BV, Proost Willemstraat 1, 6231 CV, Meerssen, The Netherlands; 2 Dusseldorf University, Dusseldorf, Germany; 3 Ernest Mario School of Pharmacy, Rutgers University, New Jersey, USA

**Keywords:** PSLT, Particles, Overload, Hazard, Risk, Lung cancer

## Abstract

**Background:**

In 2006, titanium dioxide and carbon black were classified by IARC as “possibly carcinogenic to humans” and in 2017 the European Chemicals Agency’s (ECHA) Committee for Risk Assessment concluded titanium dioxide meets the criteria to be classified as suspected of causing cancer (category 2, through the inhalation route). These classifications were based primarily on the occurrence of lung cancer in rats exposed chronically to high concentrations of these materials, as no such responses have been observed in other animal species similarly exposed. After the EU classification of titanium dioxide, it was suggested that Poorly Soluble particles of Low Toxicity (PSLTs) can be evaluated as a group.

**Main body:**

To better understand the current state of scientific opinion, we sought perspective from several international experts on topics relevant to the classification of carbon black; titanium dioxide; and, the potential future classification of PSLTs. Areas discussed included: grouping of PSLTs; the relevance of rat lung cancer responses to high concentrations of PSLTs; and, clearance overload and implications for interpretation of inhalation toxicology studies. We found there were several areas where a large majority of experts, including ourselves, agreed. These included concerns on the grouping of PSLT and the definition of clearance overload. Regarding the extrapolation of PSLT associated lung cancer in rats there were some strongly held differences, although most experts questioned the relevance when excessive exposures which overwhelm lung clearance were required.

**Short conclusion:**

Given the ongoing discussion on PSLT classification and safety, we believe it is important to re-activate the public debate including experts and stakeholders. Such an open discussion would serve to formally document where scientific consensus and differences exist. This could form the basis for design of future safety programs and safety assessments.

## Background

In 2006, titanium dioxide and carbon black were classified by IARC as “possibly carcinogenic to humans” [1] In 2017 the European Chemicals Agency’s (ECHA) Committee for Risk Assessment assessed the carcinogenic potential of titanium dioxide (TiO2) against the criteria in the Classification, Labelling and Packaging (CLP) Regulation and concluded it meets the criteria to be classified as suspected of causing cancer (category 2, through the inhalation route) [2]. The classification of these materials as possible or suspected human carcinogens has been based primarily on the occurrence of lung cancer in rats exposed chronically to high concentrations of these materials. Other species such as mice and hamsters did not develop lung cancer after chronic exposure to similarly high concentrations of these materials. Consideration is now being given to classify for carcinogenic hazard Inhaled Poorly Soluble Low Toxicity Particles (PSLT) as a class, of which carbon black and titanium dioxide are considered to be analogues. Collectively, these actions and proposals raise questions on how 30 years of inhalation toxicology and epidemiology research is being applied to ensure the best science is used to evaluate, classify and assess the risk of inhaled particulate materials.

## Main text

We have been involved in research on titanium dioxide, carbon black and other particulate materials for over 35 years. To understand the current state of scientific opinion regarding the classifications of carbon black and titanium dioxide and potential future classification of PSLTs, we sought perspective from international experts extensively involved in inhalation toxicology research; health hazard and risk assessment; and/or, regulation of the recently classified materials and other particulate materials. We consulted with 23 experts with various backgrounds (academia (*n* = 7), regulatory function with academic affiliation (*n* = 5), Industry (n = 7) and consultant (*n* = 4). We structured our conversations to cover 4 key topics: the definition of PSLTs; the relevance of the rat as a model for predicting human lung cancer hazard for inhaled poorly soluble particles; the concept of clearance overload and its influence on health risk assessment; and the expert’s opinion on recent classification decisions for carbon black and titanium dioxide. The primary focus of our discussions was on lung cancer. Below is a brief summary of what we heard from the experts along with our perspective.

### Poorly soluble low toxicity particle classification as group

A majority of experts (70%) with whom we talked saw major challenges with regulating PSLTs as a class of materials. The major concern among these experts was the lack of a clear definition of PSLTs. Key comments included:

- Such a grouping does not acknowledge the differences that exist in the inherent properties (e.g. elemental composition, surface chemistry) of materials.
- The term PSLT is used quite frequently, however, no specific definition is provided - what represents “poorly” soluble; what represents, “low” toxicity and, how should these characteristics be measured?
- A PSLT classification would need to consider particle size as it can have a dramatic effect on toxicity. There are well documented particle size-dependent differences in the behavior of titanium dioxide particles in the lung. Nano (< 100 nm diameter) sized particles show greater interstitial penetration, distributing into lung tissue and other organs not reached by larger sized particles.

A minority of experts (30%) felt a case for a group classification of PSLTs could be made. As with the detractors of grouping PSLTs, proponents noted there would need to be clear criteria (ranges and validated methodology) for solubility and toxicity. Within this minority there were experts that supported grouping in the interest of expediency to provide guidance on hazard and safety. Other experts believed any PSLT grouping strategy needed to allow for differentiating members based on potency of effect. Specific reference was made here to characteristics such as surface area or surface reactivity. Key comments included:

- This approach is similar to the historical classifications of “nuisance dust” and “particles not otherwise classified – PNOC”.
- A classification based on a PSLT grouping could provide interim guidance for managing materials until more information is available.
- It is important that within a PSLT group, members can be differentiated based on variations in bioactivity.
- Surface area reactivity may be unifying principle to distinguish PSLTs of different potency (based on mass dose) and particles with inherent toxicity.

As for the authors, we would urge the expectation be set that any time the term PSLT is used, it is accompanied by a definition. If that can’t be provided, the scientific and regulatory community should refrain from using the term. Regarding the applicability of a grouping approach, we believe that ECHA’s publication on the read-across assessment framework is instructive. ECHA describes criteria for grouping materials for health hazard and risk assessment, pre-eminent among these is “structural similarity”, other ECHA guidance is the grouped materials have common functional groups and precursors and/or breakdown products. Clearly the composition of materials referred to as PSLTs would not fit such criteria. Without a detailed definition (including chemical and physical characteristics) agreed to by the scientific community a PSLT grouping cannot be supported and would risk mis-classifying materials. There is an opportunity for the technical community to provide a scientifically sound definition of PSLTs.

### Relevance of the rat as an animal model for human hazard assessment

The rat is frequently used as an experimental model for human hazard assessment. In the 1980s and 90s several chronic inhalation studies were conducted with particles considered to have low solubility; low toxicity; and, be non-genotoxic. The materials studied included: titanium dioxide, carbon black, talc (not containing asbestiform fibers) and printer toner e.g., [1–4]. The carbon black materials tested had primary particle sizes which were nanosized; for titanium dioxide both a nanosized and larger micrometer size particle were evaluated (in different studies); talc and toner particles were large micrometer size. Lung tumors were observed in rats exposed to carbon black, titanium dioxide, and talc, but not toner particles. Where other animal species were tested (mice and hamsters) lung tumors were not observed [5]. It is the titanium dioxide and carbon black studies in rats which have been cited in support of the cancer hazard classification for these materials.

There were differing opinions among the experts consulted on the relevance of the PSLT associated rat lung cancer to human hazard. A large majority (70%) thought when lung cancer is observed only with chronic exposures producing clearance overload the relevance to humans is highly questionable. This group included experts with academic, regulatory and industry backgrounds. Key comments by this group included:

- Research has shown the rat lung response to PSLT is different from other species, this includes tumors, inflammation and clearance pathways.
- Extensive epidemiology studies have not demonstrated increased lung cancer after PSLT exposure.
- Some researchers hold on to the predictive relevance of the clearance overload associated rat lung tumors despite overwhelming evidence (including human epidemiology) to the contrary.

The balance of the experts (30%) believed a more conservative, precautionary approach was appropriate for PSLT hazard classification. As such, they believed positive lung cancer responses in the rat should be considered supportive of a carcinogen classification. Some key comments from these experts included:

- Using the rat lung cancer response to PSLT for classification represents a conservative, worst case scenario
- As seen for crystalline silica, the rat is more sensitive than other species e.g., mice. Assuming relevance is protective for humans
- Classification needs consider the totality of the data and take into account the nature of exposure (did it produce overload of normal clearance pathways), epidemiology and responses in other species.

In our opinion the rat lung tumors occurring in the inhalation studies of carbon black and titanium dioxide are of questionable relevance to human hazard. We do not argue the likely mechanism – chronic inflammation resulting in mutagenesis, and cell proliferation, is unique to rats [6]. There is evidence that chronic inflammation and cell proliferation are associated with cancer in both rats and humans. However, what is unique to the rat studies are the exposure conditions under which rats develop lung cancer; the manner in which rats vs humans clear particles from the deep lung; the overwhelming of the rat’s normal macrophage-mediated clearance processes (resulting in a disproportionate accumulation of particles); and, the severity of the rat’s lung inflammatory and proliferative response. Along with these uniqueness aspects of the rat data, other key data supporting the lack of relevance includes: the epidemiological evidence in coal miners, carbon black and titanium dioxide workers that show no excess of lung cancer in robust and worldwide cohort studies, including exposure-response analysis the absence of lung tumors and the differing tissue responses in mice and hamsters [7]; and, the rat lung tumors are largely different from those seen in human lung cancer [8].

### Overload

In 1988 Paul Morrow [9] proposed that dust overloading of the rat lung typified by a progressive reduction of particle clearance reflects a breakdown in alveolar macrophage mediated removal of particles due to loss of macrophage mobility. A consequence of this clearance overload is the disproportionate accumulation (dose) of particles in the lung with continuing exposure. There was general agreement among the experts the definition of overload put forward by Morrow is accepted and impaired macrophage clearance is mentioned almost always as a definition of overload. There was a general concern that reference to “overload” has been invoked quite freely and, at times, inappropriately to question the relevance of studies for human risk assessment. As such it was noted that regulatory documents increasingly refrain from using the term overload.

In our opinion, the overload hypothesis as proposed by Morrow was invaluable to stimulate research into the relationships between particle properties; lung clearance and lung burden; and, the outcome of inhalation studies. There should be a discrimination between clearance impairment caused by toxicity versus by volumetric overload of macrophages (PSLT). Intrinsic toxicity, such as occurs with crystalline quartz, may kill macrophages or impair their function, but this is an entirely different adverse outcome pathway (AOP) from the overload that Morrow describes. Key to extrapolating the rat lung tumor responses to carbon black or titanium dioxide are toxicokinetic factors related to exposure. We believe the concept of clearance overload must be considered in the design and interpretation of inhalation studies. Where the particulate materials are known to be of low inherent toxicity (based on in vitro and in vivo studies; chronic studies should be designed to minimize overload of normal clearance processes at the maximal exposure concentration (maximum functionally tolerated dose, MFTD). Responses occurring only when the MFTD is exceeded should not extrapolated to lower exposure levels without specific information to support relevance of the high to low dose response [10].

### Classifications

From our conversations we took away that many experts did not agree with current hazard classifications either because the rat lung cancer response was not considered relevant to humans, or the exposure conditions were extreme to what humans would experience. The absence of other supporting animal or human data indicating cancer hazard from these materials was also cited supporting this thinking. It was broadly recognized that these classifications were done appropriately within the context of existing IARC and ECHA classification guidelines.

## Conclusions

After more than 30 years of research on PSLTs, we found there are many topics  on which a majority of experts agree: the need for clear definitions of the term PSLT; concerns about grouping PSLTs for hazard classification (due to the chemical and physical diversity of materials commonly referred to as PSLTs); the widely accepted concept of clearance overload in the rat and its application in study design and interpretation; and the questionable relevance of rat lung cancer responses to human risk. There were differing points of view, in many cases of a precautionary nature with the concern being we do not fully understand the rat lung cancer mechanism. The biggest difference among experts was observed on their thoughts regarding  MoA and this provides a topic for further debate.

With this commentary we hope to re-activate the discussion on the issue of PSLT hazard and risk assessment. It would be of value to have such discussions in a public forum with the opportunity for active debate among experts and broader input and comment from stakeholders. Understanding where those with substantial knowledge and experience in the field agree and disagree could then be used to support or re-consider prior assessments; and, provide a solid foundation for future PSLT safety evaluations. Questions we believe would be worthwhile addressing include:

- Is there a scientific basis to classify PSLTs as a group? If so, how would they be defined?
- Should distinctions be made in classifying materials depending on particle size, for example nano size materials versus larger micron size [11]?
- How should maximum tolerated doses be determined for inhalation studies with poorly soluble particles and how should responses above those levels be extrapolated?
- Is the rat lung response to high doses of particles like titanium dioxide and carbon black unique to that species, or is the rat a human relevant, sensitive species;

## Abbreviations

AOP: Adverse outcome pathways
CLP: Classification, Labelling and Packaging (CLP) Regulation
ECHA: European Chemical Agency
IARC: International Agency for Research on Cancer
MoA: Mode of Action
MTD: Maximum tolerated dose
PNOC: Particles Not Otherwise Classified
PSLT: Poorly soluble low-toxicity particles
